# Latrine utilization and associated factors among people living in rural areas of Denbia district, Northwest Ethiopia, 2013, a cross-sectional study

**DOI:** 10.11604/pamj.2014.18.334.4206

**Published:** 2014-08-26

**Authors:** Yimam Tadesse Yimam, Kassahun Alemu Gelaye, Daniel Haile Chercos

**Affiliations:** 1 Department of Environmental and Occupational Health and Safety, Institute of Public Health, University of Gondar, Ethiopia

**Keywords:** Latrine utilization, hand washing facility, hygiene, sanitation

## Abstract

**Introduction:**

In Ethiopia up to 60% of the current disease burden is attributable to poor sanitation. Latrine facility coverage is increasing since Health Extension Program started, whereas less attention to quality and utilization of latrine facilities in rural Ethiopia.

**Methods:**

A community based cross-sectional study design with multistage sampling technique was employed to collect data from total of 806 households.

**Results:**

A total of 801 households with latrines were assessed for their latrine utilization status. The extent of latrine utilization among 490 (61.2%) households was satisfactory. Presence of ≤5 children (AOR: 0.379, 95% CI :( 0.196, 0.732)), job of mothers (being farmer) (AOR: 0.321, 95%CI :( 0.136, 0.757)) and rare cleaning frequency (AOR: 0.055, 95% CI :( 0.005, 0.620)) were a factor negatively associated with latrine utilization. Whereas the presence of secondary school children (AOR: 3.739, 95% CI (1.884, 7.419), educational status of mothers (AOR: 2.437, 95% CI (1.032, 5.756), latrine constructed for the second time (AOR: 2.676, 95% CI :( 1.352, 5.299)), presence of door (AOR: 3.201, 95% CI: (1.437, 7.130)), the hygienic condition of latrine (AOR: 4.327, 95% CI: (2.05, 9.134)) were factors positively associated with latrine utilization.

**Conclusion:**

Latrine utilization rate of household latrines was satisfactory. The presence of ≤ 5 years children, job of mother (farmer), educational status of mothers, presence of secondary school student, the presence of the door, frequency of latrine construction, and hygienic condition of latrine were significant predictors of latrine utilization.

## Introduction

Globally over 2.5 billion people are still without access to improved sanitation. In 2010, 15% of the population still practice open defecation [1]. In developing regions almost half the population does not have access to sanitary facilities an estimated 1.1 billion people practice open defecation, exposing themselves and their communities to major health risks [1, 2]. In sub-Saharan Africa, only 24% of the rural population was using an improved sanitation facility [3]. In Ethiopia up to 60% of the current disease burden is attributable to poor sanitation where 15% of total deaths are from diarrhoea, mainly among the large population of under-five year's children. In addition to diarrheal diseases, there is a high prevalence of worm infestations causing contributing to the high levels of malnutrition [4]. According to Ethiopia Demographic and Health Survey 2011 report 62% of households have toilet facility, 84% urban and 55% rural households. The majority of households, 82% (91% rural and 54% urban) use non-improved latrine facilities. The coverage falls short of meeting the Millennium Development Goal target. In addition to that, level of handling and utilization status of existing latrines is not known [5, 6].

Study in the Kersa district in households with latrine, the habit of hand-washing after defecation was reported to be only about 5.1%. Only 8.3% had hand washing facilities near the latrine. The habit of hand washing after defecation is significantly allied with the educational status of the respondents [7]. From an individual point of view, any latrine is better than no latrine where as from a community point of view; a high level of coverage with hygienic latrines appears to have modest health advantages [8], A study done in Hulet Ejju Enessie district showed that the duration of utilization was a strong predictor of occurrence of childhood diarrheal diseases [9].

A poor practice such as limited utilization of sanitary facilities contaminates the environment and water sources. This suggests that efforts to increase access to safe water and improved sanitation have to be joined with strategies to promote appropriate utilization of sanitary facilities [10].

Provision of sanitation facilities initiated in all parts of Ethiopia with interventions of health extension program and continued investments to increase access to safe water and improved sanitation [6]. The increasing coverage mainly achieved by campaign with less effort to change the attitudes of the households and most of the effort of health extension workers mainly focuses on new construction with less follow-up to utilization of existing latrine facilities. Therefore, it was necessary to carry out this study so as to establish baseline information on latrine utilization and factors affecting the proper utilization of latrine in Denbia district.

## Methods

### Background

The study conducted in Denbia district, which is found in North Gondar Zone, ANRS. The District is located 781 km away from Addis Ababa, the capital of Ethiopia and 216 km from Bahr Dar. From on 2007 National Housing & Population Census, the population projected by CSA for the year 2012 is 295,423 of which 147,850 are males and 147,573 are females. The district comprises 45 kebeles (40 rural and 5 urban) with a total area of 1261.96 km2 and per km2 234.1 persons live in the district [5]. According to the 2011/12 annual report of the district health office more than 94% of the households have latrine facility [11].

### Sample size

The sample size was determined by using a single population proportion formula considering the following assumptions of latrine utilization 61% [10], 95%CI, level of significance to be 5% which gives 806 study households.

### Sampling

A community based cross-sectional study collected by interviewing randomly selected sample household spouses preferably mothers in households using a structured questionnaire and by using an observational checklist from February to April 2013. Multistage sampling technique was undertaken. Eight kebeles and respective ‘gots’ were randomly selected by simple random sampling and 806 study households selected from each ‘got’ through systematic sampling.

### Operational definitions

Satisfactory latrine utilization: is a households having functional latrines, safe disposal of child faeces, no observable faeces in the compound and show at least one sign of use (foot path to the latrine not covered by grass, the latrine is smelly, spider weave in squatting hole, presence of anal cleansing material, fresh faeces in the squatting hole, and the slab is wet).

Hygienic: means no faecal matter presents inside the facility on floor or walls, which are not full and not smell bad.

Functional latrine: is a latrine that provided services at the time of data collection even if the latrine required maintenance.

Access to hand washing facilities: is availability of hand washing facilities at the entry or adjacent to the latrine.

A Child friendly feature of latrine facility: means availability of at least one of the following features; small squatting hole, lower seat and presence of potty.

### Data processing and analysis

Data was checked visually, coded and entered into Epi Info version 3.5.1 and exported to SPSS version 20.0 software package for further statistical analysis. The data analysed using bivariate and multivariate logistic regression to determine the effect of various factors on the outcome variable. The degree of association between independent and dependent variables were assessed using odds ratio with 95% confidence interval and p-value ≤0.05.

### Ethical consideration

Ethical clearance was obtained from the Institutional Review Board (IRB) of the University of Gondar, Institute of Public Health. Formal letter of cooperation was written for Denbia district Health Office. Consent of district health office and respective kebeles were obtained. Informed consent was obtained from each study subject. Any involvement in the study was carried out with the full consent of the person being interviewed. Finally after collection of the necessary data, identified problems during an evaluation process were discussed with health office so as to improve utilization of the latrine facilities in the district.

## Results

### Socio-demographic characteristics

A total of 806 households with latrines were planned to participate in the study, out of which 801 were included in the study a response rate of 99.4%. The majority of the respondents 756 (94.4%) were mothers and the remaining 45 (5.6%) were their spouse. The mean age of the respondents was 37.22 years (±10.736 SD). The majority, (98.5%) of respondents were Orthodox Christians. The majority (89.5%) of respondents were married and 602 (75%) had a family size of five or more with a mean family size of 5.95 (±1.944 SD) persons. There were ≤5 children in 420 (52.4%) households. Two hundred forty-four (39.4%) under-five children were within 3-5 years age category. Five hundred eighty-eight (73.4%) mothers and 458 (66.1%) fathers were illiterate. Six hundred eleven (76.3%) households had children attending either primary or secondary school. Six hundred ninety-three (86.5%) households were headed by fathers.

### Characteristics of latrine facilities

Types of available latrines in the district were 100% simple pit latrines. About 764 (95.4%) latrines were privately owned and the rest 37 (4.6%) was shared with their neighbours. Five hundred forty six (68.2%) of latrines was constructed two years and longer prior the study and the mean duration of having a latrine was 2.39 (±1.34) years. Most 710 (88.6%) of the respondents who had latrines explained that they were advised by extension health workers or community health agents to construct latrines. Only 42 (5.2%) respondents complained that they were imposed by other bodies like local administrators and fear of punishment.

Regarding the frequency of latrine construction 414 (51.1%) of the current latrine was the first ever latrine, while 340 (42.4%) reported that it was their second and only 47 (5.9%) noted that it was their third latrine that they had constructed. For those who built for the second time or more, the main reason attributed to the building of the new one was because the old one got flooded were 49.4%, while 38.5% damaged and only 10.9% were due to filling up of latrine.

From the functional latrines almost all of latrine slabs were made of mainly wood and mud from this 464 (76.1%) were sealed with mud and the remaining 144 (23.6%) have no properly constructed slab and only 1 cemented. About 290 (52.4%) of latrines had no cover on the squatting hole (Table 1).

**Table 1:** Characteristics of sanitation facilities of the rural community of Denbia district, May 2013 (n=609)

Characteristics of sanitation facilities	Frequency	Percent
**Distance of latrine from the house (n= 801)**		
<6 meters	36	4.5
6-50 meters	617	77.0
>50 meters	148	18.5
Latrine Location (n= 801)		
On premises	671	83.8
Outside premises	37	4.6
No premises	93	11.6
**Child friendly feature**		
Yes	347	57.0
No	262	43.0
**Latrine needs maintenance**		
Yes	332	54.5
No	277	45.5
**Presence of squat hole cover**		
Yes	290	47.6
No	319	52.4
**Presence of wall**		
Yes	463	76.0
No	146	24.0
**Presence of roof**		
Yes	369	60.6
No	240	39.4
**Presence of door**		
Yes	239	39.2
No	370	60.8

Hand washing practices were measured through proxy indicators that focus on the existence of hand washing devices near the latrine. Only 164 (26.9%) latrines have hand washing devices. Water was observed in 124 (75.6%) households and among this soap, ash was observed only in 42 (25.6%) and 23 (14.0%) hand washing stations respectively. Among all functional latrines only 65 (10.7%) of households with access to a place to wash hands that has all essential supplies (Table 1).

**Latrine Utilization** The use of the latrine was assessed based on self- reporting, and the observation of proxy indicators. The majority 695 (86.8%) of latrines was reported as used by the respondents and the rest 106 (13.2%) latrines were never used at all. Whereas based on observation 609 (76.0%) households were observed with the presence of at least one sign of use as an indication of utilization and 192 (24%) have no any sign of use. In the compound faeces were physically observed in 84 (13.8%) of households which have functional latrine. The extents of latrine utilization among 490 (61.2%) households with latrines were satisfactory.

Among the 226 households which have 3-5 years children only 20 (8.8%) children were using latrines. Of those households which have ≤5 children 133 (31.7%) households disposed their children's faeces improperly by disposing out of houses somewhere either in the backyard or in the nearby bush (Table 2). Concerning the frequency of cleaning the latrine majority 79.5% of households clean their latrine when get dirty, only 1.7% clean daily and only 400 (65.7%) latrines were founded in a hygienic condition (Table 2).

**Table 2:** Distribution of respondents by the behavioural factors in the rural community of Denbia district, May 2013

Behavioural factors	Frequency	Percent
**Latrine use by >5 years old (n=695)**		
All family members	584	84.0
Some of family members	111	16.0
**Functional latrine (n=801)**		
Yes	609	76.0
No	192	24.0
**Observable faeces in the compound (n=609)**		
Yes	84	13.8
No	525	86.2
**Latrine use by 3-5 years children (n=226)**		
Yes	20	8.8
No	206	91.2
**Disposal means of faeces of children (n=420)**		
Sanitary disposal	287	68.3
Unsanitary disposal	133	31.7

**Reasons of latrine utilization** The majority of the respondents reported to use latrines because of their understanding about the danger of excreta to health 653 (94%), to keep the environment clean 191 (27.5%), for privacy purpose, access and no other place to defecate were 50(7.2%).

### Reasons of not using the latrine

Among the reasons given by the respondents for not use of latrine facilities by adults were long live habit (60.4%) and considering open defecation comfortable (18.9%) had been the main reasons for not utilizing a latrine. The reasons given by respondents for why children not using latrines were: large squatting hole 112 (54.4%), being just a child 54 (26.2%), and floor was not safe to stand on 40 (19.4%).

### Factors affecting latrine utilization

#### Socio-demographic factors

From socio-demographic factors, marital status, educational status of the women and men, presence of primary and secondary school children in the household, job of mother and father, family income, presence of ≤5 children and presence of radio had showed a significant association with satisfactory latrine utilization in the bivariate analysis at p ≤ 0.05.

After adjusting for other confounders in the multivariate analysis, presence of ≤ 5 children, presence of secondary school student, educational status and job of mother remained significant predictors of latrine utilization. Households with secondary school children were 3.739 times more likely to utilize latrine compared to households without secondary school children (AOR: 3.739, 95% CI (1.884,7.419)). The extent of latrine utilization were 2.437 times more likely for mothers who can read and write than those unable to read and write (AOR: 2.437, 95%CI :( 1.032,5.756)).

On the other hand farmer mothers were 67.9% less likely to utilize latrine as compared to housewife's (AOR: 0.321, 95%CI :( 0.136, 0.757)). The extent of latrine utilization were 62.1% less likely for households having ≤ 5 years children than those without ≤ 5 children (AOR: 0.379, 95%CI :( 0.196, 0.732)).

#### Environmental factors

Among environmental factors distance of latrine from the house, latrine service year, squatting hole cover, presence of the door, frequency of latrine construction, need of maintenance, number of households use latrine well constructed slab and superstructure showed a significant association in the bivariate analysis at a p value ≤ 0.05 significant point. After adjusting for other confounder variables in the multivariate analysis, the only presence of door, frequency of latrine construction were significant predictors of satisfactory latrine utilization. Households who construct latrines for the second time were 2.676 times more likely to utilize their latrine compared with households having first ever latrine ((AOR: 2.676, 95% CI :( 1.352, 5.299)).

Concerning the presence of the door, households which have latrines with door were 3.201 times more likely to utilize latrine compared with latrines which have no door (AOR: 3.201, 95% CI : (1.437,7.130)).

#### Behavioural factors

Among behavioural factors frequency of cleaning and hygienic condition of latrine showed a significant association in the bivariate analysis at a p value ≤ 0.05 significant point.

After adjusting for other confounder variables in the multivariate analysis, both frequency of cleaning and hygienic condition of latrine were significant predictors of satisfactory latrine utilization. Households who clean the latrine rarely were 94.5% less likely to utilize their latrine as compared with households clean latrine daily (AOR: 0.055, 95% CI :( 0.005, 0.620)). Whereas households which have hygienic latrines were 4.327 times more likely to utilize latrine compared with latrines not hygienic (AOR: 4.327, 95% CI: (2.05, 9.134)) (Table 3).

**Table 3:** Factors associated with satisfactory latrine utilization, Denbia district, May, 2013

Factors associated with latrine utilization	Satisfactory latrine utilization		COR (95%CI)	AOR(95%CI)
	**Yes**	**No**		
**Socio-demographic factors**				
Education status of mothers				
Read & write	164(33.5)	49(15.8)	2.69(1.88,3.848)	2.437(1.032,5.756)*
Unable to read & write	326(66.5)	262(84.2)	1.00	1.00
**Job of mother**				
Housewife	430(87.8)	147(47.3)	1.00	1.00
Farmer	47(9.6)	155(49.8)	0.104(0.71,0.151)	0.321(0.136,0.757)*
Merchant & daily labourer	13(2.6)	9(2.9)	0.494(0.207,1.179)	0.223(0.049,1.012)
**Secondary school student**				
Yes	194(39.6)	84(27.0)	1.771(1.30,2.412)	3.739(1.884,7.419)**
No	296(60.4)	227(73.0)	1.00	1.00
**Presence of ≤5 children**				
Yes	214(43.7)	206(66.2)	0.395(0.294,0.531)	0.379(0.196,0.732)**
No	276(56.3)	105(33.8)	1.00	1.00
**Environmental factors**				
**Frequency of latrine construction**				
First latrine	236(48.2)	178(57.2)	1.00	1.00
Second latrine	219(44.7)	121(38.9)	1.365(1.016,1.834)	2.676(1.352,5.299)*
Third latrine	35(7.1)	12(3.9)	2.2(1.11,4.359)	1.64(0.466,5.770)
**Presence of door**				
Yes	221(45.1)	18(15.1)	4.61(2.708,7.848)	3.201(1.437,7.130)**
No	269(54.9)	101(84.9)	1.00	1.00
**Hygienic condition of latrine**				
Yes	372(75.9)	28(23.5)	10.246(6.394,16.417)	4.327(2.05,9.134)**
No	118(24.1)	91(76.5)	1.00	1.00
**Behavioural factors**				
**Frequency of latrine cleaning**				
When dirty	459(97.5)	178(93.2)	1.433(0.474,4.333)	2.949(0.535,16.249)
Rarely	3(0.6)	8(4.2)	0.208(0.037,1.163) 1.00	0.055(0.005,0.620)*
Daily	9(1.9)	5(2.6)		1.00

* Statistically significant association at p < 0.05

** Statistically significant association at p< 0.005

## Discussion

The study found that the extent of latrine utilization among 61.2% households with latrines was satisfactory which is similar to the report in Hulet Ejju Enessie (60.7%) [10]. It was also established that hygienic condition of latrine, presence of secondary school student, presence of the door, latrine constructed for the second time and educational status of mothers were significant predictors of satisfactory latrine utilization.

The majority 695 (86.8%) of latrines was reported as used by the respondents, lower than Mirab Abaya and Alaba (100%). Whereas based on proxy indicators only 76.0% of latrines were giving service which was 10.8% lower than reported as used by the respondents. The discrepancy between self report and observation might be a tendency of respondents to over report positive hygiene behaviour in the interview. This result is lower than the finding in Mirab Abaya and Alaba (93%), higher than study in Bahr Dar Zuria (62%) and similar to study in Hulet Ejju Enessie district (86.7%) [10, 12, 13].

The methods of handling of faeces of children varied among respondents: from children 3-5 age only 8.8% children who used latrines and among those households which have ≤5 children 31.7% households disposed their children's faeces improperly by disposing out of houses somewhere either in the backyard or in the nearby bush. The use of the latrine for safe disposal of children´s faeces in the present study was higher than the study kintampo, Northern Ghana (66.5%) similar when compared with the reports in Hulet Ejju Enessie. This behaviour is entirely unacceptable practice of handling faeces of children [10, 14]. Almost half of latrines were built for the second time or more, the main reason attributed to the building of the new one was the old one got flooded (49.4%) and damaged (38.5%), while only 9.7% in Alaba and 2.5% in Mirab Abaya [13]. This may indicate that the study area was more exposed to erosion or using inappropriate latrine construction materials which lead to problems during flooding, in loose soils.

Concerning hand washing facilities near latrine, only 26.9% latrines have a hand washing facility. Which is more than three times higher than the study done in Baher Dar Zuria (6.2%), Kersa (8.3%) This difference may be due to the fact that recently there has been high mobilization of the community on hygiene and sanitation which increases hand washing facility coverage of the study area. But this study result was lower than Hulet Ejju Enessie (30.8%) this difference might be due to effort difference in mobilizing the community to use hand washing facilities [8, 10, 12].

The major reasons of latrines use were their understanding about the danger of excreta to health, to keep the environment clean, privacy and convenience. This finding also supported by focus group discussion, reasons were to prevent from diseases related with excreta, to keep the environment clean and for privacy.

The most common reason for not utilizing of latrine by the households was long live habit (60.4%) and considering open defecation comfortable (18.9%). Study in Hultu Ejju Enessie identified that non functionality of latrine and staying out for work [10]. Supporting the quantitative finding, participants of the focus group discussion also mentioned long live habit, staying out for work and low awareness on use of latrine were major reasons for non utilization of latrines.

The reasons given by respondents for not using latrines by ≤5 children were: large squatting hole (54.4%), being just a child (26.2%) and (19.4%) floor was not safe to stand. This shows that latrines constructed without considering child friendly features like small squatting hole, small foot rest and presence of the potty. The findings of this study were similar to the study in Hulet Ejju Enessie district [10]. However, the use of latrines by children in the study area was not encouraging; study in Tanzania showed that children's use of latrines was associated with a significant decrease in risk of Trachoma [15].

Based on the result of multivariate analysis from socio-demographic factors as similar to the study in Hulet Ejju Enessie households with secondary school children were 3.739 times more likely to utilize latrine compared to households without secondary school children [10]. This might be due to the fact that secondary school students were more exposed to hygiene information in the school environment. The extents of latrine utilization were 2.437 times more likely for mothers who can read and write than those unable to read and write. The presence secondary school student and educational status of mother positively favoured the improvement of latrine utilization in the home environment.

Farmer mothers were 67.9% less likely to utilize latrine as compared to housewives. This might be due to the fact that housewife's have a higher chance of staying in and around their home for a long time, which have great contribution for use of household latrine. The extents of latrine utilization were 62.1% less likely for households having ≤5 years children than those without ≤ 5 children. This might be due to open defecation practice of children and improper disposal of child faeces by parents.

From the environmental factors households who construct latrines for the second time were 2.676 times more likely to utilize their latrine compared with households having first ever latrine. This utilization difference might be due to those first ever latrine users received new knowledge about sanitation recently and the habit of utilization not well developed and also some households reconstructs their latrine after the first full. Concerning the presence of the door, households which have latrines with door were 3.201 times more likely to utilize their latrine compared with latrines which have no door. This might be due to the fact that latrines which have door can insure privacy that can encourage people to use the latrine.

The association was observed between cleaning frequency and utilization of latrine. Households who clean the latrine rarely were 94.5% less likely to utilize their latrine as compared with households’ clean latrine daily. Latrines should be cleaned daily to prevent disease transmission through contact with faeces and flies and, perhaps more crucially, insanitary conditions and odour which may deter people from using them [16]. But there is no any significant difference between those clean their latrine daily and clean when dirty. Moreover, households which have hygienic latrines were 4.327 times more likely to utilize latrine compared with latrines not hygienic. The strong association between hygienic condition of latrine and utilization could be attributed to fear of contamination, odour and flies that are major problems of unhygienic latrines. Strong association also seen between improved latrine use by all household members and conducive and hygienic latrine in Tanzania [15].

## Conclusion

Based on the findings of this study we can conclude that generally the household latrines utilization rate was satisfactory and on the way to reach 82% plan of HSDP IV of Ethiopia. Whereas hand washing facility near the latrine with access to all essential supplies were very low. From this study, we conclude that presence of ≤ 5 years children, job of mother farmer] and rare cleaning of latrine were factors negatively associated with latrine utilization and educational status of mothers, presence of secondary school student, the presence of the door, frequency of latrine construction, and hygienic condition of latrine were positively associated with latrine utilization.

Based on the study attention must be given to expand latrine facilities accompanying with the hand washing device, incorporating child friendly features, superstructure which insures privacy, encouraging people to keep their latrine hygienic and Improving women's educational status and encouraging children to continue their education above primary school is very important in improving latrine utilization status of households. Therefore, in general it is recommended that integration of hygiene behavioural change with construction of sanitation facilities is crucial.
